# Effect of thioridazine on antioxidant status of HEMn-DP melanocytes

**DOI:** 10.1007/s00210-015-1144-z

**Published:** 2015-06-24

**Authors:** Michał Otręba, Artur Beberok, Dorota Wrześniok, Jakub Rok, Ewa Buszman

**Affiliations:** Department of Pharmaceutical Chemistry, School of Pharmacy with the Division of Laboratory Medicine in Sosnowiec, Medical University of Silesia in Katowice, Jagiellońska 4, PL 41-200 Sosnowiec, Poland

**Keywords:** Thioridazine, Melanocytes, Antioxidant enzymes, Hydrogen peroxide

## Abstract

Thioridazine as an antipsychotic agent was extensively used to treat various psychotic disorders, e.g. schizophrenia. However, the therapy with this drug can induce serious side effects such as extrapyramidal symptoms or ocular and skin disorders, which mechanisms are still not fully established. To gain inside the molecular mechanisms underlying thioridazine toxicity, we examined the effect of this drug on cell viability, antioxidant defence system as well as melanogenesis in normal human melanocytes. It was demonstrated that thioridazine induces concentration-dependent loss in cell viability. The value of EC_50_ was calculated to be 2.24 μM. To study the effect of thioridazine on antioxidant defence system in melanocytes, the level of hydrogen peroxide and the activities of antioxidant enzymes superoxide dismutase, catalase and glutathione peroxidase were determined. The drug in concentrations of 0.1, 0.25, 1.0 and 2.5 μM caused changes in cellular antioxidant defence system indicating the induction of oxidative stress. It was also shown that the analysed neuroleptic in concentrations of 1.0 and 2.5 μM significantly inhibited melanogenesis. The observed changes in cell viability, antioxidant defence system and melanization in normal human melanocytes after thioridazine treatment may explain an important role of reactive oxygen species as well as melanin in mechanisms involved in this drug side effects directed on pigmented tissues.

## Introduction

Thioridazine is a typical antipsychotic drug belonging to phenothiazine neuroleptics of the piperidine type. It is a mild neuroleptic, displaying sedative and antidepressant effects, which was used in the treatment of positive and negative symptoms of schizophrenia (Wójcikowski et al. 2006). Because of the fact that thioridazine treatment is associated with prolongation of the QT interval, this agent was withdrawn from the market (Buj Alvarez et al. 2007). Thioridazine also exhibits anticancer, antibacterial, antiviral, antiprotozoic as well as multidrug resistance reversal activity (Morak-Młodawska and Jeleń 2007; Rho et al. 2011). Anticancer activity of this drug results from its antiproliferative and antisurvival effects (Min et al. 2014). Thioridazine increased apoptosis in melanoma (Gil-Ad et al. 2004), endometrial (Kang et al. 2012), ovarian (Rho et al. 2011) and lymphoma (Nagel et al. 2012) cells. Due to the fact that thioridazine is a subject of many novel studies, it is important to explain molecular mechanisms underlying these drug adverse effects directed to pigmented tissues such as skin disorders, e.g. maculopapular rash, erythema multiforme, contact dermatitis, generalized urticaria, pigmentation changes and photoinduced lichenoid reaction (Arana 2000; MacMorran and Krahn 1997). It has been reported that thioridazine photosensitivity is less common than chlorpromazine but as other phenothiazine derivatives may lead to retinal pigment epithelium (RPE) damage (Drucker and Rosen 2011; Hu et al. 2002; Llambrich and Lecha 2004). The skin photosensitivity reactions are primarily seen in sun-exposed areas of the face, neck, upper chest, dorsum of the hand and lower legs and begin with a tan or brownish discoloration that progresses to a purple or slate-like metallic blue. Pigmentation changes occur usually after a long-term and/or high-dose use of phenothiazines such as chlorpromazine or thioridazine (MacMorran and Krahn 1997). Pigmentation disorders of the skin can be either hypomelanotic or hypermelanotic or may be present with a pattern of mixed hypo- and hypermelanosis (Lamer et al. 2010). The skin disorders suggest a potential role of endogenous melanin in the induction of these side effects in pigmented tissues.

Melanocytes are highly specialized cells that are not only found in the skin or eyes but are also present in the hair, inner ear, brain, lungs, heart and adipose tissue, where they produce melanin (Plonka et al. 2009; Rok et al. 2012; Tolleson 2005). The cellular antioxidant system, the first line of defence against oxidative stress, includes a number of antioxidant enzymes, such as superoxide dismutase (SOD), catalase (CAT) and glutathione peroxidase (GPx). It has been demonstrated that melanins possess superoxide dismutase activity and are able to remove reactive oxygen species (ROS), and thus may protect pigmented tissues against cellular damage (autocatalytic lipid peroxidation of membranes, lesions in DNA, cross-linkage in proteins) induced by oxidative stress (Bickers and Athar 2006; Hoogduijn et al. 2004; Otręba et al. 2012; Singh et al. 2009; Wakamatsu et al. 2008).

Previously, we have documented that another neuroleptic—chlorpromazine (Otręba et al. 2015), as well as aminoglycoside antibiotics (Wrześniok et al. 2013a,b,c), and fluoroquinolone antibiotics (Beberok et al. 2012) induce oxidative stress in normal human melanocytes, which may be a reason for many disorders.

The aim of this study was to examine the effect of thioridazine on cell viability, antioxidant defence system as well as melanogenesis in normal human melanocytes dark pigmented (HEMn-DP).

## Materials and methods

### Materials

Thioridazine hydrochloride, phosphate-buffered saline (PBS), 3,4-dihydroxy-l-phenylalanine (l-DOPA) and amphotericin B were purchased from Sigma-Aldrich Inc. (USA). Neomycin sulphate was obtained from Amara (Poland). Penicillin was acquired from Polfa Tarchomin (Poland). Growth medium M-254 and human melanocyte growth supplement-2 (HMGS-2) were obtained from Cascade Biologics (UK). Trypsin/EDTA was obtained from Cytogen (Poland). Cell Proliferation Reagent WST-1 (4-[3-(4-iodophenyl)-2-(4-nitrophenyl)-2*H*-5-tetrazolio]-1,3-benzene disulphonate) was purchased from Roche GmbH (Germany). The remaining chemicals were produced by POCH S.A. (Poland).

### Cell culture

The normal human epidermal melanocytes (HEMn-DP; Cascade Biologics) were grown according to the manufacturer’s instruction. The cells were cultured in M-254 basal medium supplemented with HMGS-2, penicillin (100 U/ml), neomycin (10 μg/ml) and amphotericin B (0.25 μg/ml) at 37 °C in 5 % CO_2_. All experiments were performed using cells in the passages 6–9.

### Cell viability assay

The viability of melanocytes was evaluated by the WST-1 (4-[3-(4-iodophenyl)-2-(4-nitrophenyl)-2*H*-5-tetrazolio]-1,3-benzene disulphonate) colorimetric assay. WST-1 is a water-soluble tetrazolium salt; the rate of WST-1 cleavage by mitochondrial dehydrogenases correlates with the number of viable cells. In brief, 5000 cells per well were placed in a 96-well microplate in a supplemented M-254 growth medium and incubated at 37 °C and 5 % CO_2_ for 48 h. Then the medium was removed and cells were treated with thioridazine solutions in a concentration range from 0.0001 to 10 μM. After 21-h incubation, 10 μl of WST-1 were added to 100 μl of culture medium in each well, and the incubation was continued for 3 h. The absorbance of the samples was measured at 440 nm with a reference wavelength of 650 nm, against the controls (the same cells but not treated with thioridazine) using a microplate reader UVM 340 (Biogenet, Poland). The controls were normalized to 100 % for each assay and treatments were expressed as the percentage of the controls.

### SOD assay

Superoxide dismutase (SOD) activity was measured using an assay kit (Cayman, MI, USA) according to the manufacturer’s instruction. This kit utilizes a tetrazolium salt for the detection of superoxide radicals generated by xanthine oxidase and hypoxanthine. One unit of SOD was defined as the amount of enzyme needed to produce 50 % dismutation of superoxide radical. SOD activity was expressed in units per milligram protein.

### CAT assay

Catalase (CAT) activity was measured using an assay kit (Cayman, MI, USA) according to the manufacturer’s instruction. This kit utilizes the peroxidatic function of CAT for determination of enzyme activity. The method is based on the reaction of the enzyme with methanol in the presence of an optimal concentration of hydrogen peroxide (H_2_O_2_). The formaldehyde produced is measured colorimetrically with 4-amino-3-hydrazino-5-mercapto-1,2,4-triazole (Purpald) as the chromogen. One unit of CAT was defined as the amount of enzyme that causes the formation of 1.0 nmol of formaldehyde per minute at 25 °C. CAT activity was expressed in nanomole per minute per milligram protein.

### GPx assay

GPx activity was measured using an assay kit (Cayman, MI, USA) according to the manufacturer’s instruction. The measurement of GPx activity is based on the principle of a coupled reaction with glutathione reductase (GR). The oxidized glutathione (GSSG) formed after reduction of hydroperoxide by GPx is recycled to its reduced state (GSH) by GR in the presence of NADPH. The oxidation of NADPH is accompanied by a decrease in absorbance at 340 nm. One unit of GPx was defined as the amount of enzyme that catalyses the oxidation of 1 nmol of NADPH per minute at 25 °C. GPx activity was expressed in nanomole per minute per milligram protein.

### H_2_O_2_ assay

H_2_O_2_ content was measured using an assay kit (Cell Biolabs, Inc., USA) according to the manufacturer’s instruction. This method is based on the ability of sorbitol to convert peroxide to a peroxyl radical, which oxidizes Fe^2+^ into Fe^3+^. Then Fe^3+^ reacts with an equimolar amount of xylenol orange in the presence of acid to create a purple product that absorbs light at maximal wavelength 595 nm. The antioxidant—butylated hydroxytoluene (BHT)—is provided to prevent further undesirable chain peroxidation. Hydrogen peroxide content in the samples was expressed in micromole per milligram protein.

### Measurement of melanin content

The melanocytes were seeded in T-25 flasks at a density of 1 × 10^5^ cells per flask. Thioridazine treatment in a concentration range from 0.01 to 2.5 μM began 48 h after seeding. After 24 h of incubation, melanocytes were washed three times with PBS and viable cells were detached with trypsin–EDTA. Cell pellets were placed into Eppendorf tubes, dissolved in 100 μl of 1 M NaOH at 80 °C for 1 h and then centrifuged for 20 min at 16,000*g*. The supernatants were placed into a 96-well microplate, and absorbance was measured at 405 nm—a wavelength at which melanin absorbs light (Ozeki et al. 1996). A standard synthetic melanin curve (0 to 400 μg/ml) was performed in triplicate for each experiment. Melanin content in thioridazine treated cells was expressed as the percentage of the controls (untreated melanocytes).

### Tyrosinase activity assay

Tyrosinase activity in HEMn-DP cells was determined by measuring the rate of oxidation of l-DOPA to DOPAchrome, according to the method described by Kim et al. (2005) and Busca et al. (1996), with a slight modification. The cells were cultured at a density of 1 × 10^5^ cells in T-25 flasks for 48 h. After 24-h incubation with thioridazine (concentration range from 0.01 to 2.5 μM), cells were washed three times with PBS, lysed and clarified by centrifugation at 10,000*g* for 5 min. A tyrosinase substrate l-DOPA (2 mg/ml) was prepared in the same lysis phosphate buffer. One hundred microlitres of each lysate were put in a 96-well plate, and the enzymatic assay was initiated by the addition of 40 μl of l-DOPA solution at 37 °C. Absorbance was measured every 10 min for at least 1.5 h at 475 nm using a microplate reader. Tyrosinase activity was expressed as the percentage of the controls (untreated melanocytes).

### MITF assay

Microphthalmia-associated transcription factor (MITF) content was measured using ELISA, an assay kit (USCN Life Science Inc, USA), according to the manufacturer’s instruction. This kit is a sandwich enzyme immunoassay for in vitro quantitative measurement of MITF providing a 96-well microplate pre-coated with a biotin-conjugated antibody specific for MITF. The colour change of the enzyme (horseradish peroxidase)–substrate (TMB) reaction was measured spectrophotometrically at 450 nm using a microplate reader. MITF content in the samples was expressed as the percentage of the controls (untreated melanocytes).

### Statistical analysis

In all experiments, mean values of at least three separate experiments (*n* = 3) performed in triplicate ± standard deviation (SD) were calculated. Statistical analysis was performed with one-way ANOVA followed by Tukey post-hoc test using GraphPad Prism 6.01 software. The significance level was established at a value of *P* < 0.05 (*) or *P* < 0.01 (**), by comparing the data with those for control (cells without thioridazine).

## Results

### The effect of thioridazine on cell viability

**Fig. 1:**
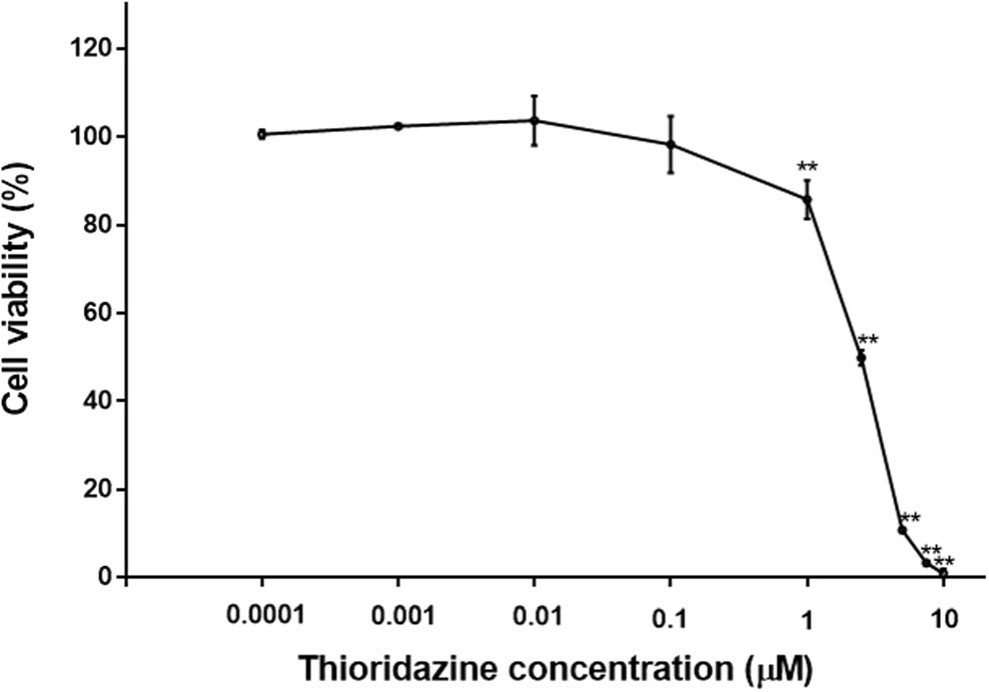
The effect of thioridazine on viability of melanocytes. Cells were treated with various doses of thioridazine (0.0001–10 μM) and examined by WST-1 assay. Data are expressed as percentage of cell viability. Mean values ± SD from three independent experiments performed in triplicate are presented. **P < 0.01 vs. the control samples

The cell viability was determined by the WST-1 test after 24-h incubation with thioridazine in a concentration range from 0.0001 to 10 μM. It has been demonstrated that the analysed drug induces concentration-dependent loss in cell viability (Fig. 1). Melanocytes treated with 1.0, 2.5, 5.0, 7.5 and 10 μM of thioridazine for 24 h lost 14.3, 50.2, 89.2, 96.7 and 99.1 % in cell viability, respectively. The value of EC_50_ (the concentration of a drug that produces loss in cell viability by 50 %) was calculated to be 2.24 μM. At lower drug concentrations (0.0001, 0.001, 0.01 and 0.1 μM), the loss in melanocytes viability was not observed.

### The effect of thioridazine on antioxidant defence system in melanocytes

To study the effect of thioridazine on reactive oxygen species metabolism in melanocytes, the activity of antioxidant enzymes and the content of hydrogen peroxide were determined. Cells were exposed to thioridazine in concentrations of 0.01, 0.025, 0.1, 0.25, 1.0 and 2.5 μM for 24 h.

**Fig. 2:**
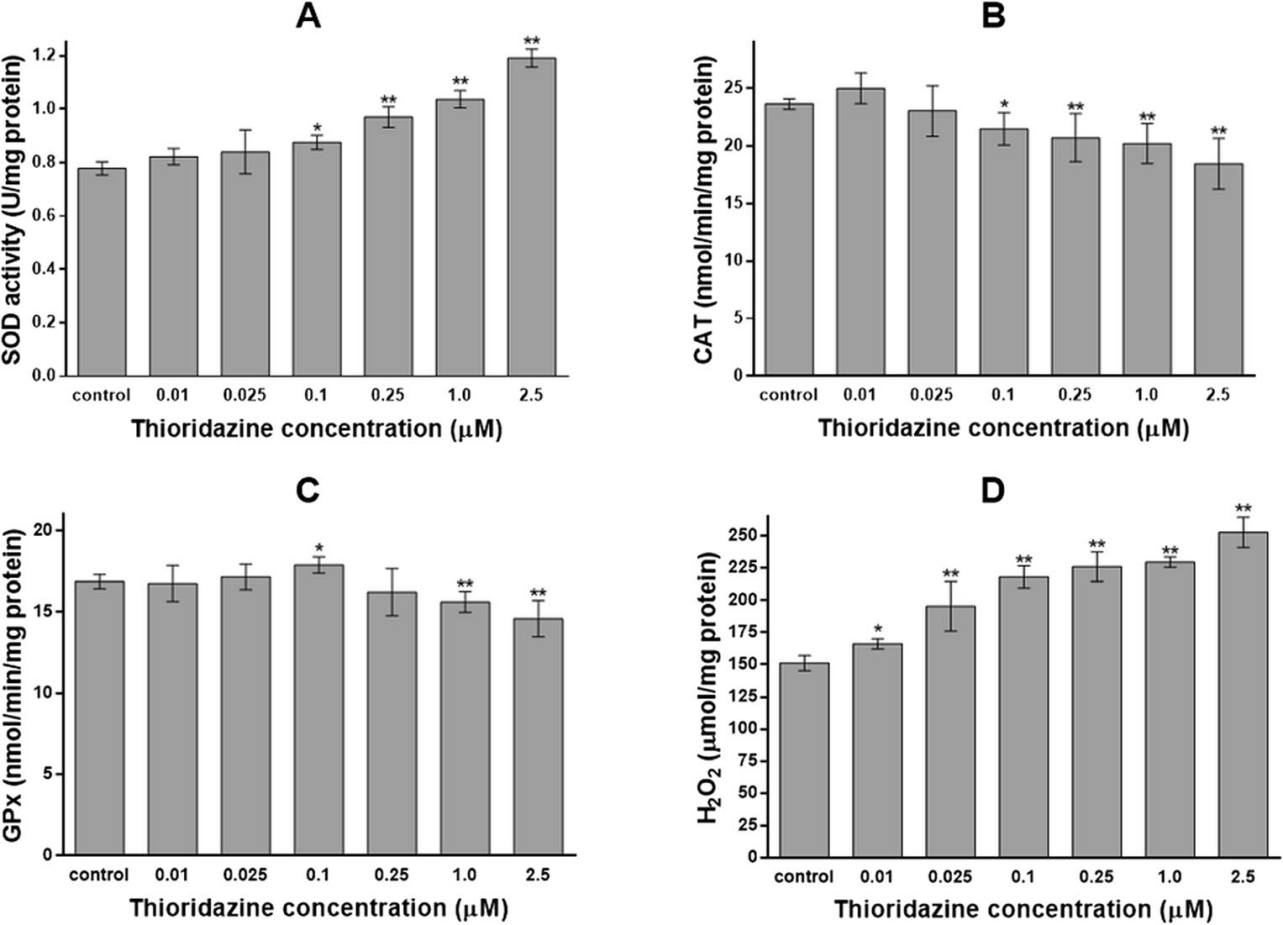
Superoxide dismutase (SOD) (**a**), catalase (CAT) (**b**) and glutathione peroxidase (GPx) (**c**) activities and hydrogen peroxide (H_2_O_2_) content (**d**) in HEMn-DP cells after 24-h incubation with 0.01, 0.025, 0.1, 0.25, 1.0 or 2.5 μM of thioridazine. Data are mean ± SD from at least three independent experiments performed in triplicate. *P < 0.05 vs. the control samples; **P < 0.01 vs. the control samples

Thioridazine raised SOD activity (Fig. 2a). After performing a calibration curve, the SOD activity was determined as 0.82 to 1.19 U/mg protein for melanocytes treated with a drug and 0.78 ± 0.06 U/mg protein for a control sample. The treatment of cells with 0.1, 0.25, 1.0 and 2.5 μM of thioridazine increased the SOD activity by 12.5, 23.9, 33.1 and 52.6 %, respectively, as compared with the controls. The analysed drug in concentrations of 0.01 and 0.025 μM had no impact on SOD activity.

After 24-h incubation with thioridazine, the intracellular CAT activity decreased (Fig. 2b). After performing a calibration curve, the CAT activity was determined as 18.45 to 24.98 nmol/min/mg protein for melanocytes treated with a drug and 23.62 ± 0.48 nmol/min/mg protein for a control sample. Treatment of HEMn-DP cells with thioridazine in concentrations of 0.1, 0.25, 1.0 and 2.5 μM decreased enzyme activity by 9.0, 12.3, 14.4 and 21.9 %, respectively. Thioridazine in concentrations of 0.01 and 0.025 μM had no impact on CAT activity.

The analysed drug modified GPx activity in melanocytes (Fig. 2c). After performing a calibration curve, the GPx activity was determined as 14.60 to 17.87 nmol/min/mg protein for cells treated with thioridazine and 16.89 ± 0.44 nmol/min/mg protein for a control sample. Treatment of melanocytes with 0.1 μM of a drug caused small increase (by 5.8 %) in GPx activity, while the concentrations 1.0 and 2.5 μM decreased the enzyme activity by 7.4 and 13.6 %, respectively. Thioridazine in concentrations of 0.01, 0.025 and 0.25 μM had no impact on GPx activity in comparison with the controls.

After 24-h incubation of melanocytes with thioridazine, the hydrogen peroxide (H_2_O_2_) content increased in a concentration-dependent manner (Fig. 2d). The H_2_O_2_ content was determined as 166.10 to 252.64 μmol/mg protein for melanocytes treated with a drug and 151.28 ± 5.92 μmol/mg protein for a control sample. The treatment of cells with 0.01, 0.025, 0.1, 0.25, 1.0 and 2.5 μM of thioridazine increased the H_2_O_2_ content by 9.8, 26.9, 44.2, 49.4, 51.7 and 67.0 %, respectively, as compared with the controls.

### The effect of thioridazine on melanization process

**Fig. 3:**
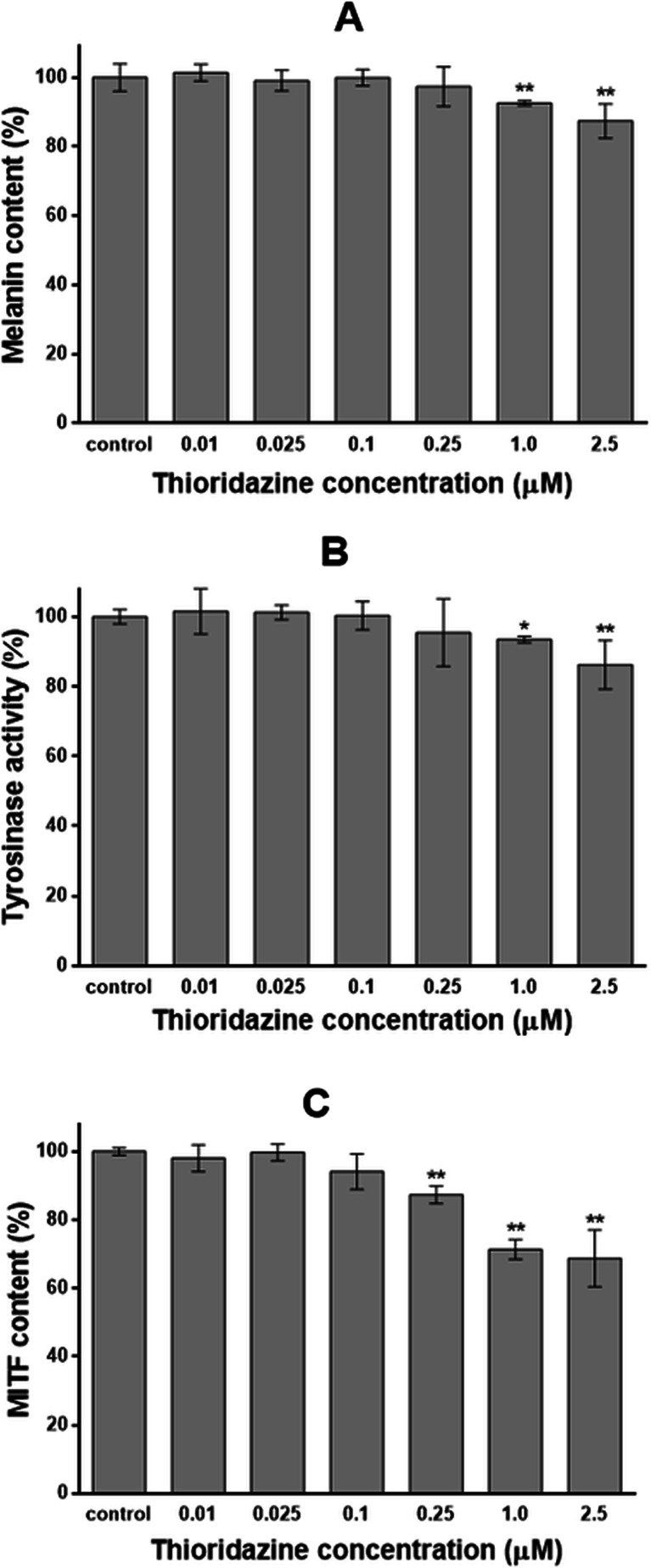
The effect of thioridazine on melanin content (**a**), tyrosinase activity (**b**) and microphthalmia-associated transcription factor (MITF) content (**c**) in melanocytes. Cells were cultured with 0.01, 0.025, 0.1, 0.25, 1.0 or 2.5 μM of thioridazine for 24 h. Data are mean ± SD from at least three independent experiments performed in triplicate. *P < 0.05 vs. the control samples; **P < 0.01 vs. the control samples

The effectiveness of melanization process was estimated by measuring the melanin content, cellular tyrosinase activity and microphthalmia-associated transcription factor (MITF) content in melanocytes treated with 0.01, 0.025, 0.1, 0.25, 1.0 and 2.5 μM of thioridazine for 24 h. After determining a calibration curve, the melanin content per cell was determined as 53.5 to 63.6 pg/cell for melanocytes treated with the analysed drug and 61.2 ± 2.39 pg/cell for a control sample. The obtained results, recalculated for culture (1 × 10^5^ cells), were finally expressed as a percentage of the controls (Fig. 3a). Treatment of HEMn-DP cells with 1.0 and 2.5 μM of a drug caused decrease in melanin content by 7.4 and 12.6 %, respectively. Thioridazine in concentrations from 0.01 to 0.25 μM had no impact on melanin content in melanocytes.

Tyrosinase activity in melanocytes treated with thioridazine decreased in a manner correlating well with the effect on melanin production (Fig. 3b). The enzyme activity was determined as 0.85 to 1.03 μmol/min/mg protein for melanocytes treated with thioridazine and 0.98 ± 0.02 μmol/min/mg for a control sample. The tyrosinase activity was decreased by 6.5 and 13.7 % for cells treated with a drug in concentrations of 1.0 and 2.5 μM, respectively, as compared with the controls. Thioridazine in concentrations from 0.01 to 0.25 μM had no impact on cellular tyrosinase activity.

After performing a calibration curve, the MITF content was determined as 0.18 to 0.26 ng/mg protein for melanocytes treated with a drug and 0.26 ± 0.01 ng/mg protein for a control sample (Fig. 3c). Treatment of HEMn-DP cells with thioridazine in concentrations of 0.25, 1.0 and 2.5 μM decreased MITF content by 12.7, 28.7 and 31.3 %, respectively. Thioridazine in concentrations from 0.01 to 0.1 μM had no impact on the cellular MITF content in comparison to the control cells.

## Discussion

Human skin is a major target for oxidative stress because of constant exposure to high levels of ROS produced by physical, chemical and biological reactions. Under normal conditions, various antioxidant enzymes, such as SOD, GPx and CAT, protect cells from the oxidative injury (Frey et al. 2007; Kalyanaraman 2013; Schallreuter et al. 2012). Melanocytes are especially sensitive to reactive oxygen species because of the low level of main enzyme responsible for degrading hydrogen peroxide—catalase (Schallreuter et al. 2008; Kim and Lee 2013). Under pathological conditions, oxidative stress arises from a serious imbalance between the free radicals and antioxidants levels and leads to modification and/or damage of lipids, proteins and DNA and thereby contributes to cellular dysfunction. Increased production of ROS after exposure to drugs and/or toxins has been linked with several major diseases such as cancer, diabetes, schizophrenia and neurodegenerative disorders (Frey et al. 2007; Kalyanaraman 2013; Mattila et al. 2007).

It is of interest that accumulation of H_2_O_2_ in melanocytes in millimolar concentrations may lead to disruption of many proteins and peptides leading to deactivation of important antioxidant enzymes including catalase, thioredoxin reductase and methionine sulphoxide reductases A and B (Schallreuter et al. 2008; Kim and Lee 2013). Sravani et al. (2009) demonstrated that high SOD levels and low levels of CAT in the skin of vitiligo patients are associated with oxidative stress in the pathogenesis of vitiligo. On the other hand, the micromolar concentrations of H_2_O_2_ cause an increase in the activity of many proteins and peptides such as tyrosinase, transcription factors (e.g. MITF, p53) as well as antioxidant enzymes (Schallreuter et al. 2008).

The aim of the present study was to investigate the effect of thioridazine on the antioxidant defence system and melanin formation in HEMn-DP melanocytes. In this study, we have used the culture of normal human epidermal melanocytes as an in vitro experimental model system.

Analysis of the effect of thioridazine on antioxidant status of HEMn-DP melanocytes demonstrated that this drug in concentrations of 0.1, 0.25, 1.0 and 2.5 μM significantly increased SOD activity and decreased CAT activity (Fig. 2a, b). The observed increase in SOD activity correlates well with the elevated level of H_2_O_2_ (Fig. 2d). The tested drug exerted a different effect on GPx activity (Fig. 2c). Thioridazine in concentration of 0.1 μM increased activity of this enzyme in comparison to 1.0 and 2.5 μM drug concentration, when decrease in GPx activity was observed. The presented decrease in CAT and GPx activity at higher drug concentrations (1.0 and 2.5 μM) may be connected with redundant cellular H_2_O_2_ level that cannot be eliminated. The activities of antioxidant enzymes and the content of H_2_O_2_ in melanocytes were normalized to viable cells which suggests that the observed changes in cellular antioxidant status may be caused by the reduced enzymes expression. Moreover, the disturbances of antioxidant defence system in melanocytes may result from the antagonistic effect of thioridazine on D_2_ receptors (Cuevas et al. 2013). Activation of dopamine D_2_ receptors regulates the production of reactive oxygen species by inhibiting pro-oxidant (NADPH oxidase) and stimulating antioxidant (CAT and SOD) enzyme activity (Cuevas et al. 2013; Iida et al. 1999). Thioridazine, which acts as a D_2_ receptor antagonist (Beaulieu and Gainetdinov 2011), can induce oxidative stress in cells. The thioridazine-induced generation of reactive oxygen species and changes in SOD and CAT activities were also stated by other authors (Rukmini et al. 2004). It was shown that thioridazine and chlorpromazine evoked oxidative stress in serum of schizophrenia patients in whom the increase of SOD and CAT activity was observed.

Because of the fact that melanin may act as a scavenger of free radicals, we have examined the effect of thioridazine on melanogenesis process. Melanogenesis is a complex, multistage biochemical pathway responsible for brownish-black eumelanins and/or reddish-yellow pheomelanin synthesis. It takes place in melanocytes, in separate cytoplasmic organelles, called melanosomes. The key regulatory and rate-limiting melanogenic enzyme is tyrosinase, which is able to catalyse the first two steps of melanogenesis: the hydroxylation of l-tyrosine and the subsequent oxidation of the intermediate l-3,4-dihydroxyphenylalanine (l-DOPA) to yield l-DOPAquinone (Hearing 2011; Liu et al. 2010; Otręba et al. 2012; Schallreuter et al. 2008). A large number of signal molecules and transcription factors regulate melanogenesis, but the major transcription factor is microphthalmia-associated transcription factor (MITF). The gene expression of main enzymes regulating melanogenesis (tyrosinase, TRP1, TRP2), cell survival, proliferation and differentiation is regulated by MITF (Kim et al. 2013; Otręba et al. 2012; Otręba et al. 2013).

The analysis of melanogenesis process in cells cultured in the presence of thioridazine demonstrates that this drug in concentrations of 1.0 and 2.5 μM decreases tyrosinase activity as well as melanin content (Fig. 3a, b). Moreover, after melanocyte treatment with thioridazine in concentrations of 0.25, 1.0 and 2.5 μM, the decrease in MITF content (Fig. 3c) was stated, which confirms the ability of this drug to inhibit melanogenesis in HEMn-DP melanocytes.

It is of interest that thioridazine has antagonistic properties for serotonin (5-HT) and dopamine (D) receptors that might be implicated in melanocyte biology. Immunocytochemical analysis of skin biopsies revealed that tryptophan hydroxylase (THP) and serotonin (5-HT) are localized primarily in normal melanocytes and in malignant melanoma cells, suggesting that the pathway for 5-HT synthesis is expressed predominantly in the melanotic cells. Serotonin can interact with specific cell surface membrane-bound receptors coupled with G proteins. 5-HT1A receptor expression was demonstrated in the basal epidermal melanocytes, 5-HT2A receptors in the epidermis of normal and eczematous human skin and 5-HT3 in the proliferative basal layer of the epidermis (Nordlind et al. 2008; Lundeberg et al. 2002). 5-HT7 receptors can stimulate and 5-HT1A receptors can attenuate adenylate cyclase activity, resulting in reducing and increasing of cyclic AMP level. Due to the fact that antagonists of 5-HT receptors may decrease the cAMP level, the reduction in expression of *TYR* and *MITF* genes may be observed (Nordlind et al. 2008; Otręba et al. 2012). Dopamine receptors are localized in the basal layer of the epidermis where they play an important role in the regulation of cell proliferation. The antagonists of D_2_ receptors reduce the cAMP level (Schallreuter et al. 2008; Slominski et al. 2012). Thus, the pharmacological properties of thioridazine with respect to its possibility to receptor binding in melanocytes should be taken into consideration in the elucidation of molecular mechanisms of thioridazine side effects in vivo. Taking into account the data published by Richtand et al. (2007) concerning the *K*_*i*_ values of dopamine and serotonin receptor binding affinity for thioridazine (*K*_*i*_ from 10 to 579 nM), it may be suggested that the analysed drug is able to D_2_ dopamine and 5-HT receptor binding in all thioridazine concentrations (from 0.01 to 2.5 μM, i.e. from 10 to 2500 nM) used in this study.

Our earlier study showed that another phenothiazine derivative, namely chlorpromazine, in lower concentrations induced melanogenesis, while changes of antioxidant status were not observed (Otręba et al. 2015). The use of that drug in higher concentrations caused similar to thioridazine significant alterations of antioxidant enzyme activity in normal melanocytes.

The present work provides the first in vitro study on the mechanisms involved in thioridazine-induced oxidative stress and pigmentation disorders using HEMn-DP cells. The obtained results may explain a potential role of thioridazine in the depletion of cellular antioxidant status leading to hypopigmentation. Our results also demonstrate that HEMn-DP cells represent a suitable cell model to study mechanisms regulating melanogenesis and antioxidant defence system in human pigmentation disorders.
